# Moving towards One Health surveillance of antibiotic resistance in France: a semi-quantitative evaluation of the level of collaboration within the national surveillance system

**DOI:** 10.1093/jacamr/dlae008

**Published:** 2024-02-01

**Authors:** Lucie Collineau, Léo Rousset, Mélanie Colomb-Cotinat, Marion Bordier, Clémence Bourely

**Affiliations:** University of Lyon—French Agency for Food, Environmental and Occupational Health and Safety (ANSES), Epidemiology and Surveillance Support Unit, Lyon, France; University of Lyon—French Agency for Food, Environmental and Occupational Health and Safety (ANSES), Epidemiology and Surveillance Support Unit, Lyon, France; Université Claude Bernard Lyon 1, Service des Etudes Interdisciplinaires en Santé, Lyon, France; VetAgro Sup, University of Lyon, USC 1223-RS2GP, Laboratory of Leptospira and Veterinary Analysis, Marcy L’Etoile, France; Santé Publique France (SPF), Infectious Diseases department, Saint Maurice, France; ASTRE, University of Montpellier, CIRAD, INRAE, Montpellier, France; CIRAD, UMR ASTRE, Dakar, Senegal; National Laboratory for Livestock and Veterinary Research, Senegalese Institute of Research in Agriculture, Dakar, Senegal; French Ministry of Agriculture and Food, General Directorate for Food, Animal Health Unit, Paris, France

## Abstract

**Objectives:**

Collaboration between surveillance programmes is the keystone of One Health surveillance and international organizations call for integrated surveillance systems to manage antibiotic resistance (ABR). In France, the ABR surveillance system covers human, animal, food and the environment sectors, but appears to be fragmented, questioning its level of integration. This study aimed to evaluate collaboration within this system and to formulate recommendations towards more integration.

**Methods:**

ECoSur, a semi-quantitative tool, was used to evaluate collaboration between surveillance programmes. A total of 31 attributes were evaluated using information from the literature and 52 interviews with surveillance actors from all four sectors. Evaluation results were visualized via three output figures displaying aspects related to governance and functionality of collaboration. Results were validated by an expert committee.

**Results:**

Overall, the French collaborative strategy for ABR surveillance was well formalized and relevant to its objectives. However, a cross-sectoral coordination body was lacking to help with its practical implementation. The environmental sector was largely uncovered, but its integration appeared necessary to meet the strategy objectives. Data sharing and joint data analyses between programmes were insufficient, mainly due to limited resources and data interoperability issues. Collaboration was operational for internal and external communication of the results. Twelve recommendations were suggested to decision makers to foster collaboration within the French surveillance system and feed future strategies against ABR.

**Conclusions:**

This first evaluation of collaboration within the French ABR surveillance system produced concrete recommendations to move towards One Health integrated surveillance. Both the approach and the findings could be of interest to other countries.

## Introduction

Antibiotic resistance (ABR) is a threat to modern health care and is recognized as one of the major public health issues in Europe and worldwide.^1^ Since antibiotic-resistant microorganisms and genes circulate within and between ecosystems, prevention and control of ABR requires integrated actions at the human–animal–environment interface. The 2015 WHO Global Action Plan on Antimicrobial resistance,^2^ and the 2017 EU One Health Antimicrobial Resistance Action Plan,^3^ underscored the need for integrated surveillance using a One Health approach to more effectively address the ABR issue. In 2021, the *Codex Alimentarius* released guidelines on integrated monitoring and surveillance of foodborne antimicrobial resistance.^4^

In France, the 2016 interministerial roadmap for controlling antimicrobial resistance^5^ has set an impulsion towards a One Health approach to surveillance, emphasizing the need to rationalize surveillance data across sectors and to promote cross-sectoral collaboration. These initiatives would complement other activities defined in the sectoral national action plans.^6–9^ However, a recent mapping and characterization of the national surveillance system in 2021 demonstrated that France has a resourceful yet complex system, made of 48 distinct programmes for surveillance of ABR, antibiotic use (ABU) and antibiotic residues in humans, animals, food and the environment.^10^ The most surveillance programmes address a single sector, and focus on either ABR or ABU, with their own surveillance protocols, databases and annual reports. This questions the extent to which current programmes are collaborating, and whether opportunities exist to facilitate integration, build synergies and mutualize surveillance activities between them. Collaboration is the primary feature of One Health integrated surveillance. Collaboration can occur at any step of the surveillance process, from governance (e.g. steering, coordination) to implementation of operational surveillance activities (e.g. sample collection, data analysis, communication of the results).

Evaluation of integrated surveillance is an emerging field of research. Various evaluation tools and frameworks have been developed in recent years, each of them having their own strengths and weaknesses.^11^ The CoEval-AMR network, funded under the Joint Programming Initiative on Antimicrobial Resistance, recently released a selection tool to guide evaluators in choosing the most appropriate framework depending on their objectives and resources.^12^ Using this selection tool, EcoSur, which has been developed for in-depth evaluation of the quality and appropriateness of multisectoral collaboration within a surveillance system,^13^ appeared as the most appropriate tool for our study.

We aimed to evaluate the degree and quality of multisectoral collaboration within the French surveillance system for ABR, ABU and antibiotic residues, and to formulate practical recommendations for improvement.

## Material and methods

### Description of the surveillance system under evaluation

The surveillance system under evaluation was the French system for surveillance of ABR, ABU and antibiotic residues in humans, animals, food and the environment, which has been described in detail in a previous publication.^10^ Briefly, this system is made of 48 distinct surveillance programmes, most of which target the human sector and to a lesser extent the animal sector, whereas only one programme covers the environmental sector (Table 1). In addition, most programmes target only ABR, or less frequently ABU, whereas only two programmes target antibiotic residues. A visual representation of the system is provided in the Supplementary material (Figure S1, available as Supplementary data at *JAC-AMR* Online).

**Table 1.** Description of the 48 programmes contributing to the French system for surveillance of ABR, ABU and antibiotic residues in humans, animal/food and the environment in 2021. Adapted from Collineau *et al*.^10^

Sector (*n* = number of programmes)	Population (*n* = number of programmes)	Number of programmes covering the target of interest^a^		
		ABR (*n* = 35)	ABU (*n* = 14)	Residues (*n* = 2)
Human (*n* = 35)	Healthcare facilities (*n* = 30)	29	3	not applicable
	Community (*n* = 23)	19	4	not applicable
	Long-term care facilities (*n* = 20)	18	3	not applicable
Animal (*n* = 12)	Diseased food-producing animals (*n* = 10)	3	7	not applicable
	Diseased companion animals (*n* = 2)	1	1	not applicable
	Healthy food-producing animals (*n* = 2)	2	none	not applicable
Food (*n* = 3)	Food of animal and non-animal origin (*n* = 1)	1	none	none
	Food of animal origin (*n* = 2)	1	none	1
Environment (*n* = 1)	Surface and ground water (*n* = 1)	none	none	1

^a^A programme may target more than one sector, population or target.

### Inclusion criteria

A collaboration was defined as ‘any collaborative activity, implemented either once, repeatedly or continuously by actors governing and/or operating within distinct surveillance programmes, and that contributes to increase the overall value of surveillance’.^13^ Collaboration both within and between sectors (i.e. human, animal, food and the environment) were included. We collected information on collaboration over the last 10 years.

### Description of EcoSur

ECoSur is a tool for standardized evaluation of the organization, operation and functionality of collaboration within a multisectoral surveillance system.^13^ It helps to identify whether collaboration, as planned and implemented, is producing the expected results given the surveillance context. Ultimately, the evaluation highlights the strengths and weaknesses of the collaboration and, where necessary, makes recommendations for improvement.

Twenty-two organizational attributes are used to assess the organization of collaboration for governance (12 attributes) and operations (10 attributes) of surveillance activities. Nine functional attributes are used to assess the key characteristics of functional and sustainable collaboration. The list of attributes is presented in Table 2. To complement these attributes, three quality indices assess the collaboration at a macro level across three major processes: management, support and realization.

**Table 2.** List of organizational and functional attributes of the EcoSur tool

Organizational attributes		Functional attributes
Governance level	Operational level	
G.1 Formalization and endorsement of the collaborative surveillance strategy G.2 Relevance of collaborative objective(s) and purpose G.3 Coverage G.4 Collaborative mechanisms for steering the system G.5 Collaborative mechanisms for coordinating the system G.6 Collaborative mechanisms for the scientific and technical support to the system G.7 Formalization and endorsement of collaborative modalities G.8 Relevance of collaborative modalities G.9 Training G.10 Performance and evaluation G. 11Information and communication G.12 Engagement	O.1 Collaboration for surveillance design O.2 Collaboration for data collection O.3 Collaboration for laboratory testing O.4 Collaboration for data management and storage O.5 Collaboration for data sharing O.6 Collaboration for data analysis and interpretation O.7 Collaboration for sharing surveillance results O.8 Collaboration for communication to surveillance actors O.9 Collaboration for external communication O.10 Collaboration for dissemination to decision makers	Stability Relevance Operationality Acceptability Resources Adaptability Inclusiveness Shared leadership System knowledge

The tool relies on a matrix developed in a Microsoft Excel sheet and including 74 evaluation criteria scored on a four-tier scale from 0 (i.e. no compliance) to 3 (i.e. full compliance). A scoring guide facilitates interpretation and harmonization of the scoring. Each score is justified by a short text that later facilitates the formulation of recommendations for improvement. The scoring of criteria first requires the completion of a data collection form summarizing the data collected from all surveillance programmes, and capturing some additional information about collaboration.

**Figure 1.**
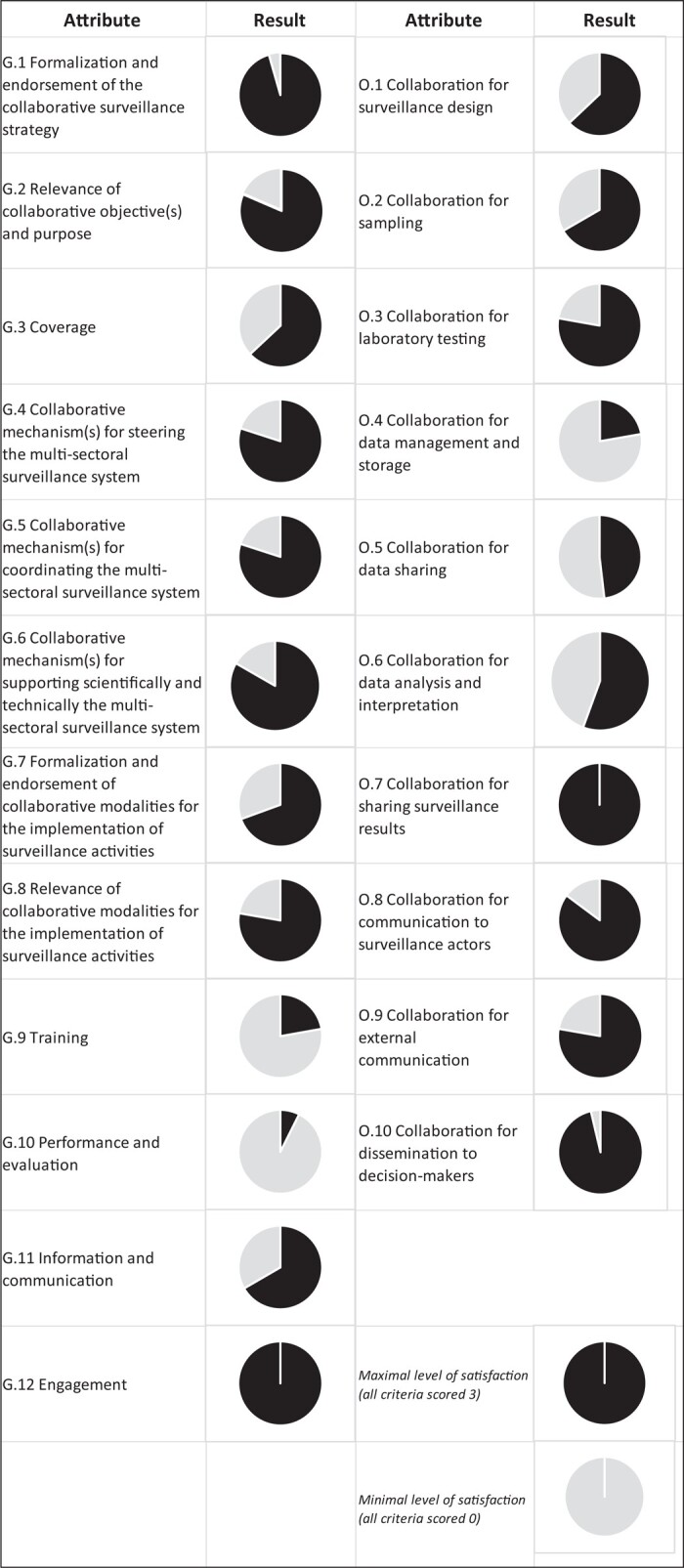
Output 1 displaying evaluation results of the antibiotic resistance surveillance system as 22 organizational attributes, France, 2021. The black part of each pie represents the level of satisfaction of the attribute compared to an ideal situation (i.e. perfect collaboration, with all criteria scoring 3). Governance attributes are displayed on the left and operational attributes on the right side of the figure.

**Figure 2.**
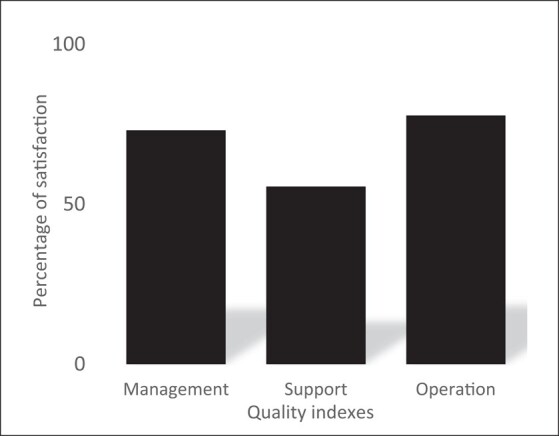
Output 2 displaying evaluation results of the antibiotic resistance surveillance system as three organizational indexes, France, 2021.

**Figure 3.**
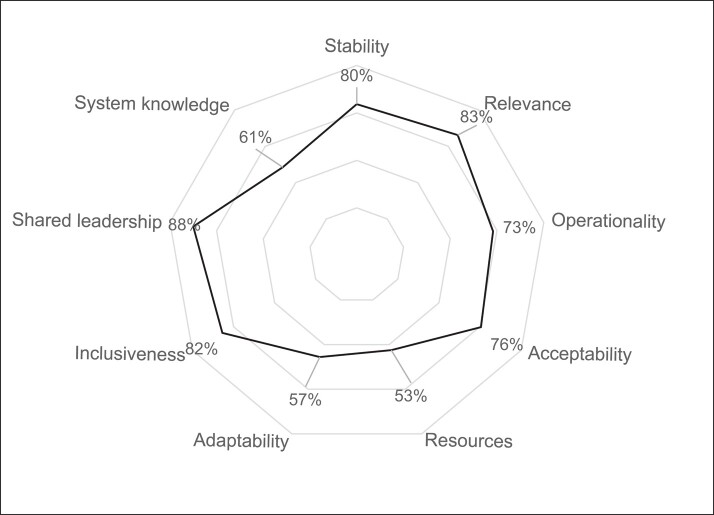
Output 3 displaying evaluation results of the antibiotic resistance surveillance system as nine functional attributes, France, 2021.

The scores obtained for the 74 criteria are grouped and used to calculate scores of the attributes, displayed using three output figures (Figures 1–3). Output 1 shows 22 organizational attributes, including 12 attributes focusing on governance aspects (e.g. collaborative strategy, resources, steering and coordination activities) and 10 focusing on operational aspects (e.g. surveillance design, data sharing, data analysis and dissemination of the results). Output 2 shows three organizational indexes related to management, support and operation of collaborative activities. Output 3 shows nine functional attributes defining an appropriate collaboration (e.g. relevance, acceptability and shared leadership). A detailed description of EcoSur is provided in Bordier *et al.*^13^ and in the following webpage: https://survtools.org/wiki/surveillance-evaluation/doku.php?id=quality_of_the_collaboration.

### Data collection

Out of the 48 programmes identified in the mapping of the French system for ABR surveillance, five were excluded from our study because ABR surveillance represented only a minimal part of their activity.^10^ Hence, 43 programmes were included in this evaluation.

For each surveillance programme, data were collected using information available from the literature (e.g. annual reports, programme website). To complete and validate these data, 36 interviews with the programme coordinator(s) were conducted, based on an interview guide (Supplementary material, Table S1) addressing the elements of the data collection file. For each collaboration, we collected information related to the context (i.e. scientific, political, economic context that shaped collaboration), governance (i.e. strategy, coverage, governance mechanisms, resources/funding, training, monitoring and evaluation, information) and operations (i.e. collaborative activities, including frequency and actors involved).

To get a broader sense of current collaboration between surveillance programmes, 15 additional key informants with core expertise in ABR surveillance in the four sectors, operating within the French surveillance system and/or having a good understanding of the overall surveillance system, were also interviewed. Their profile and expertise are provided in Supplementary material, Table S2. The interview guide was adjusted to these actors to capture aspects related to governance and operations of collaborations at the level of the entire surveillance system, as well as strengths and weaknesses of the surveillance system influencing on collaborations and potential new collaborations to be encouraged (Supplementary material, Table S3). Interviews were conducted between March and May 2021 using online videoconferencing.

### Data analysis and validation

Using information gathered in the data collection file and form, the scoring of the 74 criteria was performed by the evaluation team made of two experts from the human sector and two experts from the animal and food sectors. Of the four evaluators, three were internal to the ABR surveillance system and one was external. Scores were then discussed and validated during a 1-day workshop with a group of 19 experts with long-term expertise in surveillance or policy-making related to ABR, ABU and antibiotic residues surveillance in the human, animal, food and environmental sectors. Minor edits were made to the scoring where needed.

The workshop outcomes, together with the EcoSur matrix outputs, were used to formulate practical recommendations for the improvement of collaboration within the surveillance system. These recommendations were ordered according to their decreasing impact to foster the overall integration of the surveillance system in the short- and mid-term.

### Ethics

Written consent to participate was obtained from every interviewee. Ethical approval was obtained from the department of legal affairs of the French Agency for Food, Environmental and Occupational Health & Safety.

## Results

### Evaluation of organizational attributes

The output of EcoSur for organizational attributes applied to the French ABR surveillance system is displayed in Figure 1. With regards to the governance of ABR surveillance, the French surveillance system scored high in terms of formalization and endorsement of the collaborative strategy (G1) and relevance of its collaborative objectives (G2) were 96% and 81%, respectively. Indeed, the national collaborative strategy, as described in the interministerial roadmap^5^ and the national action plans,^6–9^ was well formalized and relevant with the broader context, including the political, socio-economic and epidemiological situation, as well as the European and international recommendations. In addition, ABR was clearly recognized as a major public health issue in the different sectors. Different documents systematically formalized the collaborations involving more than two programmes. However, in terms of collaborative mechanisms for coordinating the multisectoral surveillance system (attribute G5, 80%), a cross-sectoral coordination body was lacking, while considered necessary to facilitate the implementation of the national collaborative strategy. Furthermore, while stakeholders appeared fully engaged in various national working groups dedicated to surveillance (attribute G12, 100%), some sectors (e.g. the environmental sector) and disciplines (e.g. social sciences) appeared under-represented, notably in steering activities (i.e. those activities aiming to define the vision and objectives of the ABR surveillance system, attribute G4, 80%). Training was judged insufficient (attribute G9, 22%) mainly because of the lack of training courses in One Health surveillance of ABR. Additionally, the attribute related to monitoring and evaluation (G10) was scoring very low (7%) due to the absence of regular evaluations of the level of integration or collaboration within the surveillance system, this study being the first of this kind.

Regarding the organization of collaboration at the operational level, collaboration existed for surveillance activities implemented at the end of the surveillance process, such as sharing surveillance results (attribute O7, 100%), internal and external communication (attributes O8 and O9, 85% and 78%, respectively), as well as dissemination of the results to decision makers (attribute O10, 96%). One concrete example in this area is the publication of a One Health Antibiotic Resistance brochure coordinated by Santé Publique France, summarizing the key results from the major national surveillance programmes into a single document and released annually at the occasion of World Antimicrobial Resistance Awareness Week.^14^ Conversely, little collaboration existed for surveillance activities at the beginning of the surveillance process, such as design of surveillance protocols (attribute O1, 63%), data management (attribute O4, 22%), data sharing (attribute O5, 48%) or data analysis (attribute O6, 56%), where collaboration mainly occurred via time-limited research projects. While joint data analysis was partly conducted at the European level under the umbrella of the Joint Inter-Agency Antimicrobial Consumption and Resistance Analysis,^15^ no similar initiative was in place at the national level in 2021.

### Evaluation of organizational indices

The French ABR surveillance system scored relatively high for the operation index (78%), reflecting that collaborative activities were appropriate and in line with the collaborative strategy, and that the results produced were relevant. Similarly, the management index was satisfactory (73%): collaborative mechanisms for steering and coordination were generally well formalized and surveillance actors were fully committed to the roles and responsibilities assigned to them. Conversely, the system scored lower (56%) for the support index, in relation to a lack of various elements facilitating proper functioning of collaboration, such as training and resources.

### Evaluation of functional attributes

Five out of nine functional attributes of collaboration obtained a high score (>80%) (Figure 3). Major strengths of the French ABR surveillance system were the shared leadership and inclusiveness, with the appropriate, relevant and active involvement of stakeholders in steering and scientific committees. This contributed to the relevance and acceptability of the collaborative strategy. Despite a general lack of resources, collaboration was evaluated as stable since the collaborative strategy was clearly formalized and approved by the stakeholders. Operationality scored relatively high as several collaborative activities were already in place, e.g. joint communication. However, several operational aspects were lacking for the system to be fully operational and to respond to the needs of the stakeholders, e.g. ABR surveillance in the environment, the use of common ABR indicators across sectors, as well as a shared database to facilitate data integration and joint analysis. System knowledge scored low due to the lack of liability of the overall surveillance system, as well as the lack of institutional memory and some collaborative activities occurring via informal channels or within closed subgroups. The adaptability of the system scored very low (57%) due to a lack of systematic evaluation of collaboration.

### Recommendations for improved collaboration

Building from the results of the EcoSur evaluation, 12 recommendations for improved collaboration within the French ABR surveillance system were formulated and shared with decision makers from the human, animal, food and environmental sectors (Table 3).They aim to address the major gaps identified in our evaluation, namely the need for better operational coordination to implement the national collaborative strategy, the lack of surveillance in the environmental sector, as well as the need for improved interoperability and joint analysis of surveillance data. Better legibility of the surveillance system, as well as increased visibility and impact of the One Health ABR brochure, were also part of the top five recommendations.

**Table 3.** Recommendations for improved collaboration within the system for surveillance of antibiotic resistance, France, 2021

Recommendation number (by decreasing order of priority)	RECOMMENDATIONS	JUSTIFICATIONS
**1**	Create a One Health operational body for national coordination of surveillance of ABR, grouping together the actors working at the operational level and contributing to facilitate collaboration between programmes. Its role could be to improve data interoperability and to propose harmonized surveillance protocols (e.g. data formats, standards, and interpretation criteria for antimicrobial susceptibility testing), to facilitate joint communication of the results between sectors and between ABU/ABR, and to work towards mutualization of surveillance resources.	The current interministerial roadmap for controlling antimicrobial resistance lacks operationality. Several technical difficulties have been identified that limit data interoperability between sectors/programmes: different geographical and temporal granularity, lack of harmonization of surveillance methods (e.g. heterogeneity of standards and interpretation criteria used for antibiotic susceptibility testing, etc.). These specificities hinder data integration efforts and interoperability of the data.
**2**	Reinforce surveillance in the environment, with full-scale implementation of environmental surveillance, beyond surface and groundwater. Surveillance could be expanded to community and hospital wastewater, coastal seas and farm environments. In addition to the impact on human and animal health, the impact of environmental ABR on ecosystems should also be considered.	The environmental sector is currently largely uncovered. Structured national surveillance of antibiotic residues in the environment is limited to surface and groundwater. In addition, collected data are not sufficient to monitor the impact of environmental ABR on ecosystems, which is one of the objectives of the surveillance.
**3**	Create a national cross-sectoral working group dedicated to (i) the definition and interpretation of common indicators across sectors and programmes, and (ii) the implementation of integrated data analysis across sectors and programmes, inspired from the European joint inter-agency antimicrobial consumption and resistance analysis (JIACRA) reports.	There is little or no joint analysis and interpretation of surveillance data at the system level. The interministerial roadmap (action 30) recommended defining common indicators to measure antibiotic resistance and exposure jointly in humans, animals, and the environment, but it is not effective at this stage. Only part of the French data is transmitted to the European level and analysed jointly across sectors via the JIACRA report 22. However, there is currently no such ‘JIACRA-like’ joint analysis of the surveillance data available in France.
**4**	Improve the legibility of the French ABR surveillance system: 4.1. Clarify the role of each actor and programme; 4.2. Develop a unique and sustainable entry point/dashboard to access reports, websites and data from the various French surveillance programmes.	The characterization and mapping of the French surveillance system^10^ showed a complex system with several gaps and overlaps between surveillance programmes. The surveillance data produced by the various programmes were generally accessible, but very dispersed and with poor visibility.
**5**	Strengthen joint communication of surveillance data from the three sectors: 5.1. Reinforce the dissemination and impact of the One Health Antibiotic Resistance brochure coordinated by Santé Publique France and better precise its target audience. 5.2. Envisage co-coordination of the One Health Antibiotic Resistance brochure across sectors. 5.3. Where needed, develop other joint communication materials to better respond to the needs of the various actors and end-users.	Joint communication of surveillance data mainly occurs in November during the World Antimicrobial Resistance Awareness Week, and should be better coordinated between institutions and implemented more regularly. The One Health ABR brochure coordinated and financed by Santé Publique France ^14^ is one of the most successful initiatives today in terms of joint and multisectoral communication across ABR surveillance programmes in France. However, its impact and visibility currently appear limited, due to its rather restricted dissemination. The target audience of the One Health ABR brochure should also be clarified.
**6**	Facilitate the exchange of phenotypic and molecular data between programmes 6.1 Establish mechanisms to facilitate data exchange (e.g. common portal or data warehouses). 6.2 Deploy sequencing to more surveillance programmes with access to bacterial strains, either by pooling resources between programmes or by strengthening molecular analyses for programmes already collecting strains.	There is currently little exchange of phenotypic or molecular data, and this is mainly done on an *ad hoc* and project-based basis. There is no common portal or national-wide data warehouse that brings together surveillance data, either phenotypic or molecular data, from different sectors and which is accessible to the different surveillance actors. Confidentiality and ownership of data can also be a barrier to collaboration. For programmes with access to bacterial strains, some have the skills and resources (including computing capacity) to sequence strains routinely, and others do not.
**7**	Expand the scope of existing surveillance in human and animal sectors: 7.1. Strengthen surveillance of the healthy carriage of bacteria/resistance genes in humans. 7.2. Improve surveillance of antibiotic use (ABU) in companion animals. 7.3. Promote surveillance in French overseas territories.	In human health, surveillance primarily focuses on clinical isolates from diagnostic laboratories but data on ABR carriage are sparse. In animal health, surveillance of antibiotic use in companion animals is still limited (overall sales data). The overseas territories are poorly covered by existing national surveillance programmes. A regional surveillance programme exists in the Indian Ocean as part of the One Health partnership in the IndianOcean (known as dP One Health OI) but is not connected to the other national programmes.
**8**	Strengthening joint steering activities: 8.1. Broaden stakeholder participation in the various national steering committees to other disciplines (e.g. social sciences, economics) and to the environmental sector; 8.2. Better define the roles and responsibilities of the actors involved in the steering committees (e.g. roles and responsibilities of the pilots of the actions of the national action plans); 8.3. Sustain resources dedicated to steering and monitoring of the interministerial roadmap and national action plans.	The main stakeholders and end-users are involved in the steering committee of the interministerial roadmap, but not all of them (e.g. absence of some stakeholders from the environmental sector). Moreover, some disciplines (e.g. social sciences, economics) are not represented either in this committee or in the sectoral steering committees. The roles of the pilots of the actions of the interministerial roadmap and the sectoral plans in the animal sector (EcoAntibio) are well defined, but their level of responsibility with regard to what is and is not produced needs to be clarified. An acute critical lack of human and financial resources for the follow-up of some plans was identified.
**9**	Strengthen communication from the government on the One Health aspects, to the stakeholders involved in operational implementation in the field: local and regional stakeholders. In particular, more emphasis should be made on the organization (existence and role of steering committees).	Government actions in favour of intersectorality (e.g. the interministerial roadmap for controlling ABR) are not well known to the professional actors involved in ABR activities. In general, the actions are known to the actors directly involved at the central level, but little at regional/local levels.
**10**	Sustain and formalize collaborative initiatives: 10.1. Facilitate, through dedicated financial schemes, the sustainability of collaboration between programmes, particularly those initiated as part of research projects; 10.2 Include (cross-sectoral) collaboration between programmes in the terms of reference of the national surveillance programmes. This would contribute to dedicating part of the national surveillance programmes budget to the implementation of collaboration for surveillance.	In the majority of cases, there are no specific resources for establishing collaboration between surveillance programmes, whether for data sharing, joint analysis or communication, or scientific and technical support between programmes. The budgets allocated for national surveillance in the human sector are determined for 5 years, and do not take into account the potential additional resources needed to establish collaboration, including data sharing Most surveillance programmes receive funding for routine operational activities, but funding is limited for advanced data analysis.
**11**	Create/reinforce initial and continuous training in One Health approaches for those stakeholders involved in antibiotic resistance and antibiotic use.	Training courses in One Health approaches already exist but they are not always known; nor are they specific to antibiotic resistance. Conversely, education in the field of antibiotic resistance is not necessarily One Health (cross-sectoral).
**12**	Regularly assess the level of collaboration within the ABR surveillance system in France: 12.1. Establish performance indicators dedicated to the level and quality of collaboration; 12.2. Plan external and internal evaluations of collaboration for ABR surveillance; these could be integrated into the evaluations of surveillance programmes already carried out.	The establishment of collaboration between surveillance programmes has not been comprehensively evaluated and valued to date.

## Discussion

### Summary of the main findings

This study represents the first attempt to evaluate the degree and quality of collaboration within the French ABR surveillance system. Overall, the evaluation showed that the national One Health collaborative strategy was well formalized, relevant and approved by all actors. Some operational collaborative activities were already in place, e.g. internal and external communication of the results.

However, some discrepancies were identified between the dimensions (e.g. sectors, disciplines) included in the multisectoral surveillance system and the objectives of the national strategy. Despite the recognized impact of ABR on ecosystems, the environmental sector was largely uncovered, as already shown previously.^10^ Some disciplines (e.g. social sciences, economics) were not represented in the major surveillance steering committees.

Furthermore, collaborative activities upstream of the surveillance process, e.g. the design of surveillance protocols, data sharing and joint data analysis were limited at this stage, primarily because of a lack of coordination and sustainable resources. However, the pooling of these upstream activities would notably improve data harmonization and facilitate cross-sectoral data analysis and interpretation of the results, beyond joint communication of the results. It would also be in line with the national collaborative strategy requiring the use of common indicators across sectors and programmes.

Building from the evaluation results, 12 practical recommendations for improving collaboration were formulated and shared with decision makers from the human, animal, food and environmental sectors to feed in particular the revision of the interministerial roadmap for controlling ABR.^16^

### Strengths and limitations of the study

A major strength of this study was the broad and comprehensive approach we used, exploring collaboration between programmes in all the dimensions of the surveillance system. This was facilitated by a previous mapping study^10^ that helped to decipher the complexity of the national surveillance system. Combining data from the literature together with interviews, we are confident we fully captured all existing collaborative efforts within the surveillance system up to 2021. Additionally, our evaluation results were validated through a dedicated workshop gathering experts from the four sectors and from several institutions involved at different levels of the surveillance system. This allowed us to capture different perspectives on the objectives and utility of surveillance, and to produce realistic and practical recommendations.

However, this study also had limitations. First, we focused on the degree and quality of collaboration, which is only one of the multiple facets of One Health surveillance. Other related aspects were only partly covered. For example, we assessed data interoperability without exploring in details the data management systems (e.g. data formats, thesaurus, etc.), which was beyond the scope of this study. Another important aspect that was poorly covered in our study was the added value or impact of collaboration. This aspect could be further explored using other complementary evaluation frameworks or tools, e.g. ISS-AMR^17^ or OH-EPICAP.^18^

### Feedback on the use of EcoSur

Overall, EcoSur proved helpful to support a thorough evaluation of collaboration within a complex and fragmented surveillance system, including both governance and operational aspects. The output figures nicely summarized the scores of the numerous criteria into a limited number of evaluation attributes, hence facilitating communication of the results to an external audience. The justification of each score facilitated the formulation of practical recommendations for improvement. The tool is generic and can easily be adapted to various systems and contexts.

As previously reported,^11^ the terminology being used in EcoSur is, however, very specific and complex. Specific training was needed to get acquainted with the tool before use. In addition, as EcoSur requires detailed data to be collected on each collaborative activity, the size and complexity of the French ABR surveillance system made data collection a time-consuming process.

While EcoSur facilitates the visualization of the level of collaboration within the surveillance system, the tool does not cover the visualization of the surveillance system itself. A tool inspired by social network analysis,^19^ where each node of the network represents a surveillance programme, and the edges are the collaborations between them, would nicely complement EcoSur.

### Perspectives

Some of the recommendations formulated here should shortly be addressed as part of two large national meta-networks launched in November 2021. The meta-network PROMISE^20^ that gathers 25 networks and 42 academic stakeholders, is currently developing a joint data warehouse for ABR surveillance that will help to design and conduct cross-sectoral analysis. Also under the scope of PROMISE, a national network for environmental surveillance of ABR is setting up, to improve structured surveillance of ABR in the environment. In addition, the meta-network ABRomics-PF^21^ aims to build a platform for ABR multi-omics One Health data sharing. These two initiatives appear to be excellent opportunities to further facilitate collaboration between surveillance programmes. They also demonstrate that France has a dynamic ABR surveillance system with ever-evolving collaboration.

Complementing the previous mapping of the French ABR surveillance system conducted in 2021,^10^ as well as an in-depth investigation of the main drivers for collaboration between surveillance programmes,^22^ this evaluation of the degree and quality of collaboration is an important step of a broader approach to support the development of integrated surveillance in a One Health context. We believe that this stepwise approach will also inspire other countries considering a gradual transition towards One Health surveillance of ABR, as our methodology can easily be transferred to other countries and situations.

## Supplementary Material

dlae008_Supplementary_Data

## Supplementary data

Figure S1 and Tables S1 to S3 are available as Supplementary data at *JAC-AMR* Online.

## References

[1] Murray CJ, Ikuta KS, Sharara F et al Global burden of bacterial antimicrobial resistance in 2019: a systematic analysis. Lancet 2022; 399: 629–55. 10.1016/S0140-6736(21)02724-0 35065702 PMC8841637

[2] World Health Organization . Global Action Plan on Antimicrobial Resistance. 2015. Available at: https://www.emro.who.int/health-topics/drug-resistance/global-action-plan.html 10.7196/samj.9644 26242647

[3] European Commission . A European One Health Action Plan Against Antimicrobial Resistance (AMR). 2017. Available at: https://health.ec.europa.eu/system/files/2020-01/amr_2017_action-plan_0.pdf

[4] FAO, WHO . Guidelines on integrated monitoring and surveillance of foodborne antimicrobial resistance—CXG 94-2021. 2021. Available at: https://www.fao.org/fao-who-codexalimentarius/thematic-areas/antimicrobial-resistance/en/. Accessed December 20, 2022.

[5] Interministerial Committee on Health . Interministerial Roadmap for controlling antimicrobial resistance. 2016. Available at: https://solidarites-sante.gouv.fr/IMG/pdf/interministerial_amr_roadmap_en.docx.pdf. Accessed December 11, 2020.

[6] French Ministry of Solidarity and Health . Stratégie nationale 2022-2055 de prévention des infections et de l’antibiorésistance [2022-2055 national strategy for preventing infections and antibiotic resistance]. 2022. Available at: https://sante.gouv.fr/IMG/pdf/national_strategy_for_preventing_infections_and_antibiotic_resistance_2022-2025_pdf

[7] French Ministry of Agriculture and Food Sovereignty . Ecoantibio2: the French national plan for the reduction of the risks of antimicrobial resistance in veterinary medicine 2017-2021. 2022. Available at: https://agriculture.gouv.fr/telecharger/119352

[8] Ministry of Ecological Transition and French Ministry of Solidarity and Health . Un environnement, une santé—4e plan national santé environnement (PNSE4). [2020-2024 National Environmental Health Plan (PNSE4). 2022. Available at: https://www.ecologie.gouv.fr/plan-national-sante-environnement-pnse

[9] Collineau L, Bourély C, Rousset L et al Towards One Health surveillance of antibiotic resistance: characterisation and mapping of existing programmes in humans, animals, food and the environment in France, 2021. Euro Surveill 2023; 28: 2200804. 10.2807/1560-7917.ES.2023.28.22.2200804 37261729 PMC10236929

[10] Sandberg M, Hesp A, Aenishaenslin C et al Assessment of evaluation tools for integrated surveillance of antimicrobial use and resistance based on selected case studies. Front Vet Sci 2021; 8: 620998. 10.3389/fvets.2021.620998 34307513 PMC8298032

[11] Rüegg SR, Antoine-Moussiaux N, Aenishaenslin C et al Guidance for evaluating integrated surveillance of antimicrobial use and resistance. CABI One Health 2022; 2022: ohcs20220007. 10.1079/cabionehealth.2022.0007

[12] Bordier M, Delavenne C, Nguyen DTT et al One Health surveillance: a matrix to evaluate multisectoral collaboration. Front Vet Sci 2019; 6: 109. Available at: https://www.frontiersin.org/article/10.3389/fvets.2019.00109. Accessed February 10, 2022. 10.3389/fvets.2019.00109 31106210 PMC6492491

[13] Santé publique France . Prévention de la résistance aux antibiotiques : une démarche « Une seule santé ». 2022. Available at: https://www.preventioninfection.fr/wp-content/uploads/2022/11/2022_Synthese_Une_Seule_Sante_Web_BAT_vdef.pdf

[14] ECDC, EFSA, EMA . Third joint inter-agency report on integrated analysis of consumption of antimicrobial agents and occurrence of antimicrobial resistance in bacteria from humans and food-producing animals in the EU/EEA: JIACRA III 2016-2018. EFSA J 2021; 19: e06712. 10.2903/j.efsa.2021.6712 34221148 PMC8243991

[15] French Government . Feuille de route interministérielle 2023—2033. Prévention et réduction de l’antibiorésistance, lutte contre la résistance aux antimicrobiens. 2023. Available at: https://sante.gouv.fr/IMG/pdf/feuille_de_route_interministerielle_antibioresistance.pdf

[16] Aenishaenslin C, Häsler B, Ravel A et al Evaluating the integration of One Health in surveillance systems for antimicrobial use and resistance: a conceptual framework. Front Vet Sci 2021; 8: 12. 10.3389/fvets.2021.611931 PMC8024545 33842569

[17] Tegegne HA, Bogaardt C, Collineau L et al OH-EpiCap: a semi-quantitative tool for the evaluation of One Health epidemiological surveillance capacities and capabilities. Front Public Health 2023; 11: 1053986. 10.3389/fpubh.2023.1053986 37250092 PMC10213933

[18] Martínez-López B, Perez AM, Sánchez-Vizcaíno JM. Social network analysis. Review of general concepts and use in preventive veterinary medicine. Transbound Emerg Dis 2009; 56: 109–20. 10.1111/j.1865-1682.2009.01073.x 19341388

[19] PROMISE . Promise—Professional community network on antimicrobial resistance. 2023. Available at: https://amr-promise.fr/. Accessed April 21, 2023.

[20] Institut Français de Bioinformatiques . ABRomics—a numerical platform on antimicrobial resistance to store, integrate, analyze and share multi-omics data. 2023. Available at: https://www.france-bioinformatique.fr/en/news/abromics-a-numerical-platform-on-antimicrobial-resistance-to-store-integrate-analyze-and-share-multi-omics-data/. Accessed April 21, 2023.

[21] Bourély C, Rousset L, Colomb-Cotinat M et al How to move towards One Health surveillance? A qualitative study exploring the factors influencing collaborations between antimicrobial resistance surveillance programmes in France. Front Public Health 2023; 11: 1123189. Available at: https://www.frontiersin.org/articles/10.3389/fpubh.2023.1123189. Accessed July 11, 2023. 10.3389/fpubh.2023.1123189 37497029 PMC10367569

[22] ECDC, EFSA and EMA . JIACRA III—Antimicrobial consumption and resistance in bacteria from humans and animals. 2021. Available at: https://www.ecdc.europa.eu/en/publications-data/third-joint-interagency-antimicrobial-consumption-and-resistance-analysis-report. Accessed August 13, 2021.

