# Supplementary material for: Moving towards One Health surveillance of antibiotic resistance in France: a semi-quantitative evaluation of the level of collaboration within the national surveillance system
Source: JAC Antimicrob Resist. 2024 Feb 1;6(1):dlae008. doi: 10.1093/jacamr/dlae008 (PMC10833649; doi:10.1093/jacamr/dlae008)
Supplement: dlae008_Supplementary_Data [file dlae008_supplementary_data.docx]

**Supplementary material**

Figure S1. Mapping of the existing surveillance programmes for antibiotic resistance (ABR), antibiotic use (ABU) and antibiotic residues in humans, animals/food and the environment in France in 2021. Adapted from Collineau et al. (2023)^1^


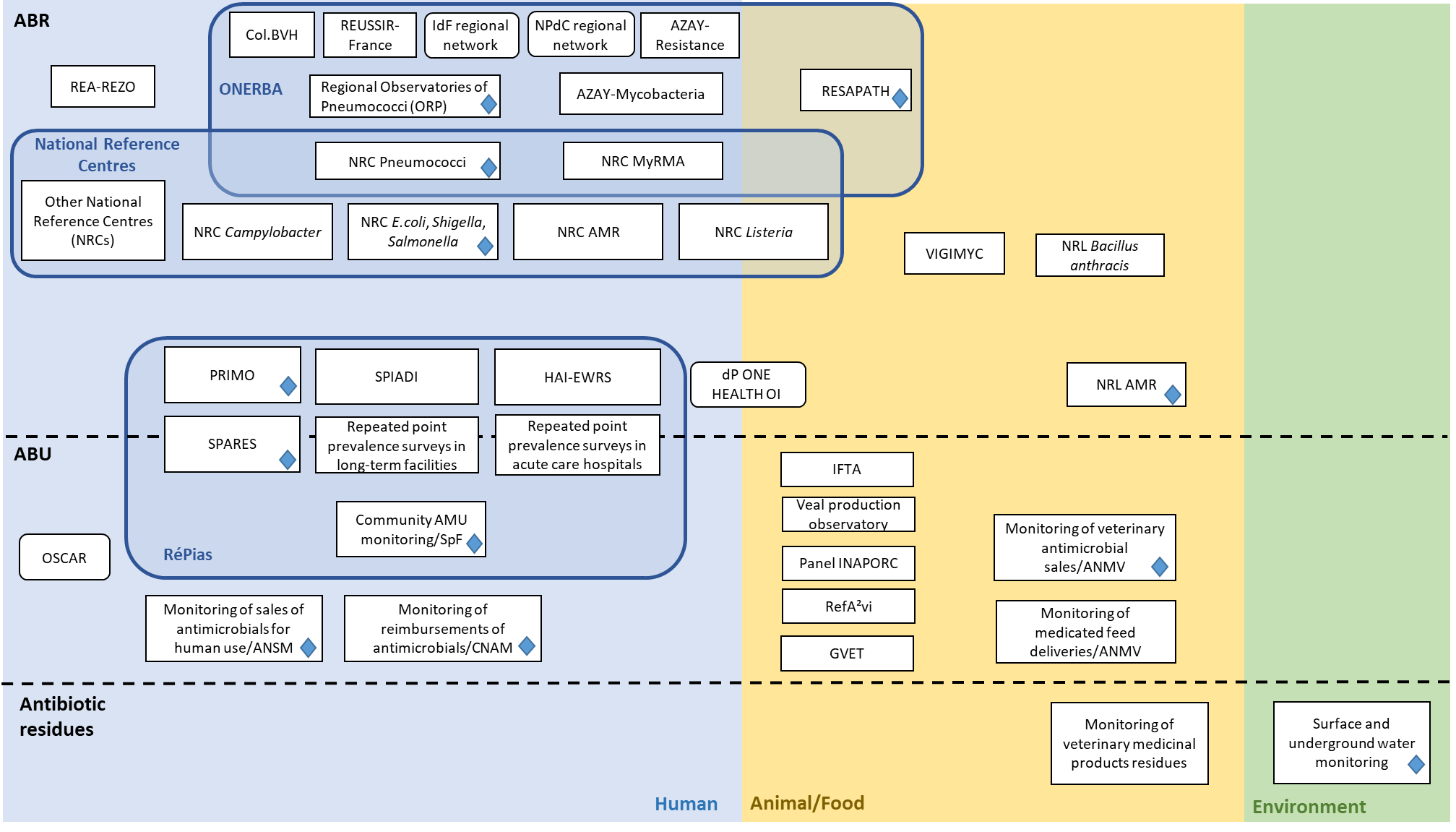


Legend: White boxes: French surveillance programmes (straights corners = national; rounded corners = regional).

Blue diamonds: programmes contributing to the annual joint One Health Antibiotic Resistance brochure coordinated by Santé publique France at the occasion of the annual World Antimicrobial Awareness Week (12 programmes involved).

Table S1. Guide used for interviews of the programmes’ coordinators for surveillance of antibiotic resistance, antibiotics use, and antibiotics residues, France, 2021

| **PART I** |
| --- |
| **Role and activities of the interviewee in the surveillance programme** |
| What is your background/discipline? |
| What is your current position? |
| At which level are you working (national, regional, local)? |
| What is your role in the surveillance programme? |
| What activities do you achieve in the surveillance programme? |
| **PART II** |
| **Collaboration between the programme evaluated and other antibiotic resistance (ABR) surveillance programmes** (questions were adapted according to the information already collected elsewhere in the literature) |
| 1. Is the programme you coordinate involved in collaboration with other surveillance programmes. If yes, which ones? 2. For which reason(s) was the collaboration established? What are the collaborative objectives? What is the ultimate goal of the collaboration for surveillance? 3. Could you please describe the organisation and functioning of the collaboration? 4. At which step(s) of the surveillance process (steering, coordination, scientific and technical support, laboratory analyses, data storage and management, etc.) does collaboration occur? To which extent/level is collaboration operation at each of these steps? 5. Who are the surveillance stakeholders involved in collaboration? 6. What are the concrete activities implemented for each of these surveillance activities? 7. Is collaboration formalized in a document? If yes, which document? By whom was this document approved? 8. Is collaboration operating as expected? Do you know if collaborative objectives are being met? 9. Are specific resources (human, financial, material) allocated to support this collaboration? 10. Is collaboration influenced by external drivers (e.g. regulatory, political, etc)? Are you aware of the interministerial roadmap for controlling ABR? 11. What are the strengths of your surveillance programme that are contributing to facilitate collaboration? 12. What are the weaknesses of your surveillance programme that are hindering appropriate collaboration? 13. What are possible ways of improvement to facilitate further collaboration? 14. What are your expectations when engaging in collaboration with another surveillance programme? 15. Have you identified other programmes it would be relevant for your programme to collaborate with? 16. In your opinion, what activities can you build upon to kick start collaborations with other surveillance programmes or sustain new ones? Conversely, what are the constraints preventing the establishment of new collaboration? 17. Have you in mind other surveillance programmes that are missing today and that could be developed to improve ABR surveillance in France? What would be their added value? 18. Are you aware of other collaborations between ABR surveillance programmes in France? If yes, which ones? |

Table S2. Profile and expertise of the 15 key informants interviewed as part of this study

| **Respondant #** | **Sector** | **Profile** | **Role/Contribution** |
| --- | --- | --- | --- |
| K1 | Human | Ministry | Officer leading One Health activities and antibiotic resistance (ABR) research through incitative policy |
| K2 | Human/Environment | Academia | Research leader in ABR environmental surveillance |
| K3 | Human | Ministry | Member of ministerial delegatation for ABR |
| K4 | Human | Academia | Member of the committee for national heathcare-associated infection and ABR in humans |
| K5 | Human | Academia | Member of the French national observatory for epidemiology of bacterial resistance to antibiotics |
| K6 | Animal/Food | Public health agency | Researcher involved in ABU/ABR surveillance in animals/food and JIACRA analysis at EU level |
| K7 | Human | Academia | Research leader in ABR environmental surveillance and national / European One Health projects on ABR |
| K8 | Human | Academia | Research leader in ABR in human sector and national / European One Health projects on ABR |
| K9 | Environment | Ministry | Officer leading incitative policy in environmental sector for ABR research and control strategies |
| K10 | Human | Academia | Researcher involved in the coordinator of the French national priority research programme for ABR |
| K11 | Animal | Public health agency | Researcher involved in animal ABR surveillance at national level |
| K12 | Animal/Food | Ministry | Officer leading incitative policy in animal / food sector for ABR research and control strategies |
| K13 | Environment | Public health agency | Researcher involved in thecoordination of the French national network for ABR surveillance in the environment |
| K14 | Environment | Ministry | Officer leading ABR control strategies in the environment |
| K15 | Environment | Public health agency | Officer involved in regional surveillance of antibiotic residues in surface water |

Table S3. Guide used for interviews of the key informants for surveillance of antibiotic resistance, antibiotics use, and antibiotics residues, France, 2021

| **PART I** |
| --- |
| **Activities of the key informant contributing to the structuring of ABR surveillance in France** |
| What is your current position? |
| What is your background/discipline/area of expertise? |
| Which of your various activities help to structure the surveillance of antibiotic resistance in France?  (For example, to support existing programmes, encourage the development of new surveillance programmes, or ensure that they work more/better together) |
| **PART II** |
| **One Health-ness of the current ABR surveillance system and changes needed** |
| 1. Do you think the sectors covered by the ABR surveillance system as it exists in France today are relevant and sufficient? What are the reasons for this? What can be done about it? 2. Do you feel that the resources dedicated to monitoring antibiotic resistance in France are sufficient? What additional resources should be allocated to monitoring antibiotic resistance (human, financial, material)? In what way (specific allocation, increase in the overall budget of the CNRs/NRLs, etc.)? 3. Can you think of any other surveillance programmes (currently non-existent, or present in other countries) that could be set up to improve antibiotic resistance surveillance in France and explain why? 4. Can you think of any other actors or institutions that should be involved in monitoring antibiotic resistance in France? How do you think we should go about integrating them into the surveillance system? 5. Do you think it would be useful to formalise the One Health approach for surveillance of antibiotic resistance in France? If so, what could be the objective(s) of a One Health system for surveillance of antibiotic resistance in France? 6. In your opinion, does the implementation of a One Health approach to surveillance of antibiotic resistance in France require dedicated resources? If so, what resources (human, material, financial) and for what types of activity (data sharing, etc.)? |
| **Collaboration within the French system for surveillance of antibiotic resistance in France** |
| 1. What regular/ongoing collaboration do you know of between antibiotic resistance surveillance programmes in France? 2. In your opinion, are current collaborations between antibiotic resistance surveillance programmes sufficient? What are the reasons for this? What could be done about it? 3. In your opinion, do current collaborations have the appropriate resources (human, financial, material)? What can be done about this? 4. In your opinion, are current collaborations sufficiently formalised? Does this influence their results? 5. In your opinion, what are the key factors in the smooth running and development of collaboration between surveillance programmes? 6. In your opinion, what are the obstacles to the smooth running and implementation of collaboration between antibiotic resistance surveillance programmes? 7. Can you think of any collaboration between surveillance programmes yet to be established? 8. In which sector(s) should collaboration be established/strengthened as a priority? 9. In your opinion, what factors should be taken into account to initiate and sustain these new collaborations between antibiotic resistance surveillance programmes in France? |

**References**

1. Collineau L, Bourély C, Rousset L, *et al.* Towards One Health surveillance of antibiotic resistance: characterisation and mapping of existing programmes in humans, animals, food and the environment in France, 2021. *Euro Surveill* 2023; **28**: 2200804.
